# Morphometric and physical characteristics of Indonesian beef cattle

**DOI:** 10.5194/aab-66-153-2023

**Published:** 2023-06-06

**Authors:** Yudi Adinata, Ronny Rachman Noor, Rudy Priyanto, Lucia Cyrilla, Pita Sudrajad

**Affiliations:** 1 Research Center for Animal Husbandry, Research Organization for Agriculture and Food, National Research and Innovation Agency, Bogor 16911, Indonesia; 2 Faculty of Animal Science, IPB University, Bogor 16680, Indonesia

## Abstract

To assess their potential for beef production, this study describes the morphometrics, physical traits, and body weight of 1034 Indonesian beef cattle from eight breeds, namely Bali, Rambon, Madura, Ongole Grade, Kebumen Ongole Grade, Sasra, Jabres, and Pasundan. An analysis of variance in addition to cluster, Euclidean distance, dendrogram, discriminant function, stepwise linear regression, and morphological index analyses were performed to describe the differences in traits among breeds. The morphometric proximity analysis revealed two distinct clusters with a common ancestor, where the first cluster included Jabres, Pasundan, Rambon, Bali, and Madura cattle and the second included Ongole Grade, Kebumen Ongole Grade, and Sasra cattle, with an average suitability value of 93.20 %. This showed that the classification and validation methods can be used to distinguish breeds. The most important factor in estimating body weight was the heart girth circumference. Ongole Grade cattle had the highest cumulative index, followed by Sasra, Kebumen Ongole Grade, Rambon, and Bali cattle. A cumulative index value >3 can be used as a threshold for determining the type and function of beef cattle.

## 1 Introduction

Indonesia has many indigenous and local cattle breeds. Based on statistics from the year 2021m the national cattle population is $17\;times\;10^{6}E$ head (BPS, 2021), and at least 23 cattle breeds have been registered with the Food and Agriculture Organization (FAO) of the United Nations via the Domestic Animal Diversity Information System (DAD-IS). Bali and Ongole Grade are the local cattle breeds with the largest populations and widest distributions (BPS, 2011). These breeds have adapted to the tropical climate and harsh conditions of Indonesia (Sutarno and Setyawan, 2016). However, a recent study indicated that their existence is threatened by indiscriminate crossbreeding (Agus and Widi, 2018; Nugroho et al., 2021). The growing popularity of exotic breeds has increased the possibility of local cattle extinction because the genetic diversity of cattle needs to be maintained to provide genetic material for their future development (Bett et al., 2013). Indigenous and local cattle play important roles in improving livestock productivity (Rojas-Downing et al., 2017).

A thorough understanding of farm animal characteristics is required for the effective management of their genetic resources (Nyamushamba et al., 2017). Morphometrics and physical appearance are important traits in breeding programs (FAO, 2012). These traits are used for genetic conservation, in addition to determining whether animals are suitable for selection, and to improve their production and realize their potential. Felius et al. (2014) stated that distinct, breed-specific morphological traits are always noticeable, while molecular genetic variance does not provide comprehensive information about adaptation. Animal traits allow us to determine the best uses for each breed and are thus of interest to beef producers (Hocquette et al., 2014).

While morphometric studies have examined several Indonesian cattle breeds, there is inadequate information on the relationship between body size and the physical appearance of indigenous and local cattle in Indonesia. Both morphometric and physical measurements are very useful for assessing whether animals are suitable as beef producers and for optimizing their potential. Based on these considerations, this study assessed the morphometric and phenotypic performance of indigenous and local cattle in Indonesia to provide information on the potential of beef-producing cattle and inform the selection criteria of breeding programs.

## 2 Materials and methods

### 2.1 Animals

**Figure 1:**
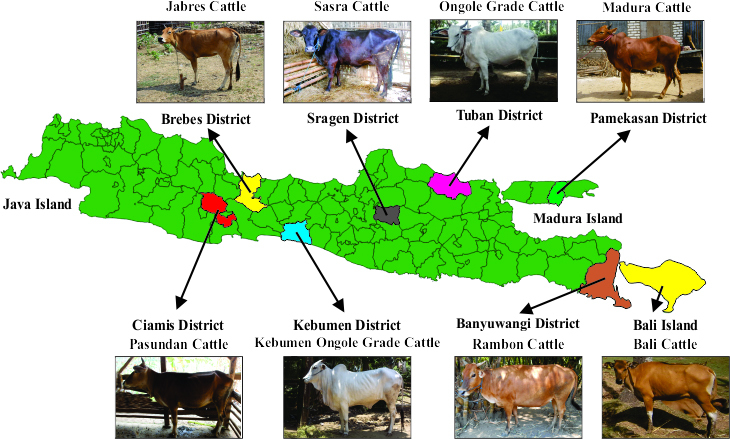
Pictures of sampled Indonesian cattle populations and the map of the cattle breeding tract. All animal pictures are from a private collection. The map of the Java, Madura, and Bali islands was created using QGIS 3.24 (QGIS Development Team, 2022).

This study examined 1034 adult cows (age range of 5–10 years; estimated from the $I_{4}$ dentition status) belonging to eight cattle breeds in Indonesia. Cattle in this age range are considered to have complete bone and muscle cell development (Lawrence et al., 2001). The animals included 136 head of Bali cattle from Bali, 100 head of Rambon cattle from the Banyuwangi province, 139 head of Madura cattle from the Pamekasan district, 139 head of Ongole Grade cattle from the Tuban district, 112 head of Sasra cattle from the Sragen district, 167 head of Kebumen Ongole Grade cattle from the Kebumen district, 139 head of Jabres cattle from the Brebes district, and 102 head of Pasundan cattle from the Ciamis district. Images of the cattle breeds and their geographic locations are presented in Fig. 1. Maps of the Java, Madura, and Bali islands were created in QGIS 3.24 (QGIS Development Team, 2022).

### 2.2 Data collection

**Figure 2:**
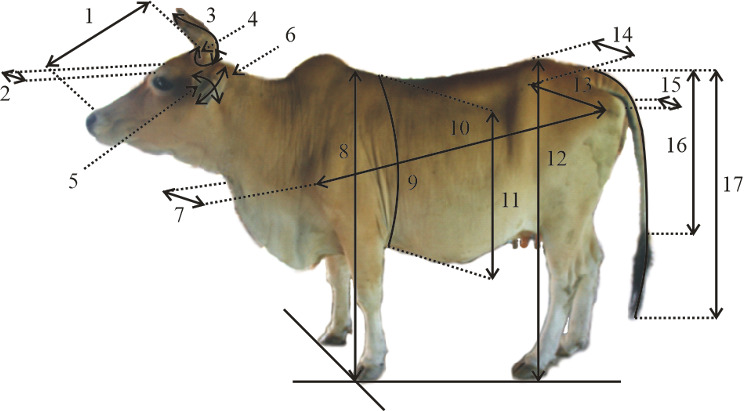
Biometric measurement points of cattle. Note: (1) head length (HdL), (2) head width (HdW), (3) horn length (HrL), (4) horn circumference (HrC), (5) ear length (ErL), (6) ear width (ErW), (7) chest width (CsW), (8) withers height (WtH), (9) heart girth circumference (HtGC), (10) body length (BdL), (11) heart girth (HtG), (12) hip height (HpH), (13) rump length (RmL), (14) hip width (HpW), (15) rump width (RmW), (16) tail length (TiL), and (17) tail length up to switch (TiLS).

Surveys and direct observations were performed from July 2019 to February 2020. Body weight (BW) was measured using a digital scale (MK Cells, USA) with a maximum capacity of 1000 kg and an error of 0.5 kg. Body measurements were made in centimeters (cm) using a calibrated ruler, measuring tape, and calipers. To avoid variation, the measurements were made by the same technician. Based on guidelines for the phenotypic characterization of cattle (FAO, 2012), 17 morphometric characters were measured, including head length (HdL), head width (HdW), horn length (HrL), horn circumference (HrC), ear length (ErL), ear width (ErW), chest width (CsW), withers height (WtH), heart girth circumference (HtGC), body length (BdL), heart girth (HtG), hip height (HpH), rump length (RmL), hip width (HpW), rump width (RmW), tail length (TiL), and tail length up to switch (TiLS). The cows were in the parallelogram standing position when measured. Figure 2 shows the biometric measurement points. In total, 12 physical characteristics/traits of the cattle were assessed, namely forehead coat color (FCo), dewlap size (DSz), hump size (HmSz), horn color (HrCo), horn orientation (HrOr), switch color (SCo), muzzle shape (MSp), muzzle color (MCo), coat color (CCo), hoof color (HoCo), hoof shape (HoSp), and vulva color (VCo). Table 1 summarizes the value ranges for each trait.

**Table 1:** The variables in the physical observations of cattle and their value ranges.

Physical variables	Color variation	Scores
Forehead coat color (FCo)	Black, gray, brown, red, color combination, and degradation white	1 to 6
Dewlap size (DSz)	Small, medium, large	1 to 3
Hump size (HmSz)	Absent, small, medium, large	0 to 3
Horn color (HrCo)	Black, gray, brown, red, color combination, and degradation white	1 to 6
Horn orientation (HrOr)	Tips pointing forward, backward, laterally, upward, downward, or various combinations	1 to 6
Switch color (SCo)	Black, gray, brown, red, color combination, and degradation white	1 to 6
Muzzle shape (MSp)	Sharp, moderately sharp, flat	1 to 3
Muzzle color (MCo)	Black, gray, brown, red, color combination, and pale white	1 to 6
Coat color (CCo)	Black, gray, brown, red, color combination, and degradation white	1 to 6
Hoof color (HoCo)	Black, gray, brown, red, color combination, and degradation white	1 to 6
Hoof shape (HoSp)	Sharp-angled hooves, moderately angled	1 to 2
Vulva color (VCo)	Black, gray, brown, red, color combination, and degradation pale white	1 to 6

### 2.3 Statistical analyses

Descriptive statistics (mean and standard deviation) and an analysis of variance were used to examine the differences in the morphometric data among cattle breeds. For each morphometric trait, the least square means and standard error were calculated using the general linear model procedure. Through hierarchical cluster analysis, animals with relatively homogeneous morphometric and physical data were grouped together, and the Euclidean distance between the groups was determined. The groupings are presented hierarchically using a dendrogram. To determine the appropriateness of the groupings, discriminant function analysis was performed (Acciaro et al., 2020).

Stepwise linear regression analysis was used to determine the best BW estimation model (Vanvanhossou et al., 2018). The coefficient of determination ( $R^{2}$ ) was calculated to determine the reliability of the model, where values closer to 1 indicate better model performance (Moriasi et al., 2007). The root mean square error (RMSE) was then computed; the lower the value, the more accurate the model estimate (Chai and Draxler, 2014). The statistical analyses were performed using SPSS software (SPSS Inc., USA).

The seven morphological indexes for beef cattle of Alderson (1999) were estimated, namely the height slope index (HSI), width slope index (WSI), length index (LI), depth index (DI), foreleg length index (FLI), balance ( B ), and cumulative index (CI). These indexes were calculated using Eqs. (1)–(7), respectively:

1HSI=WtH-HpH,2WSI=HpWCsW,3LI=BdLWtH,4DI=HtGWtH,5FLI=WtH-HtG,6B=RmL×HpWHtG×CsW,7CI=BWaveragedBWofcattlebreed+LI+B.

## 3 Results

First, 17 morphological parameters were evaluated. Table 2 gives the least square means and associated standard errors for the morphometric measures and BW of the eight cattle breeds. Most of the breeds studied varied significantly in body size. Bali, Jabres, Madura, and Pasundan cattle had the lowest morphometric values, while Sasra, Ongole Grade, Kebumen Ongole Grade, and Rambon cattle had the highest values. The order of the average live weight, from smallest to largest, was as follows: Madura, Bali, Pasundan, Jabres, Rambon, Ongole Grade, Sasra, and Kebumen Ongole Grade cattle. Table 3 gives the frequency data for the physical characteristics and shows the phenotype variation among and within cattle breeds. A value of 100 % indicates a highly uniform characteristic unique to a specific cattle breed.

**Table 2:** The least square means with standard deviations of cattle morphologies and body weight for Indonesian cattle.

Traits	Bali	Rambon	Madura	Ongole	Sasra	Kebumen	Jabres	Pasundan
				Grade		Ongole Grade		
	( n=136 )	( n=100 )	( n=139 )	( n=139 )	( n=112 )	( n=167 )	( n=139 )	( n=102 )
HdL (cm)	36.15 ${}^{a}$ ± 1.42	40.96 ${}^{b}$ ± 2.73	40.15 ${}^{c}$ ± 1.61	48.44 ${}^{d}$ ± 2.84	46.15 ${}^{e}$ ± 2.31	50.89 ${}^{f}$ ± 2.95	40.29 ${}^{c}$ ± 2.25	41.76 ${}^{g}$ ± 2.31
HdW (cm)	16.88 ${}^{a}$ ± 1.10	18.02 ${}^{b}$ ± 1.03	18.06 ${}^{b}$ ± 1.22	19.54 ${}^{c}$ ± 1.43	21.70 ${}^{d}$ ± 1.63	21.19 ${}^{e}$ ± 1.26	16.97 ${}^{a}$ ± 1.24	17.63 ${}^{f}$ ± 1.26
HrL (cm)	17.80 ${}^{a}$ ± 3.43	21.13 ${}^{b}$ ± 7.28	8.33 ${}^{c}$ ± 2.63	19.90 ${}^{b}$ ± 6.02	19.73 ${}^{d}$ ± 8.80	20.94 ${}^{b}$ ± 5.76	16.51 ${}^{a}$ ± 7.06	15.43 ${}^{a}$ ± 6.75
HrC (cm)	15.07 ${}^{a}$ ± 1.70	14.57 ${}^{a}$ ± 2.41	11.28 ${}^{b}$ ± 1.61	19.45 ${}^{c}$ ± 2.82	13.50 ${}^{e}$ ± 3.50	17.00 ${}^{f}$ ± 2.00	13.08 ${}^{d}$ ± 2.71	13.71 ${}^{d}$ ± 2.90
ErL (cm)	22.56 ${}^{a}$ ± 2.08	22.36 ${}^{a}$ ± 1.74	20.70 ${}^{b}$ ± 1.42	25.58 ${}^{c}$ ± 1.73	24.53 ${}^{d}$ ± 2.79	27.72 ${}^{e}$ ± 2.27	20.87 ${}^{b}$ ± 2.51	20.88 ${}^{b}$ ± 2.68
ErW (cm)	13.91 ${}^{a}$ ± 0.87	14.10 ${}^{a}$ ± 1.64	12.50 ${}^{b}$ ± 0.88	14.35 ${}^{a}$ ± 0.90	15.74 ${}^{c}$ ± 1.39	15.78 ${}^{c}$ ± 1.06	12.94 ${}^{b}$ ± 1.10	12.64 ${}^{b}$ ± 1.17
CsW (cm)	31.63 ${}^{a}$ ± 3.01	33.26 ${}^{b}$ ± 3.95	30.77 ${}^{a}$ ± 3.05	34.66 ${}^{b}$ ± 3.09	41.30 ${}^{c}$ ± 4.63	37.40 ${}^{d}$ ± 3.59	30.89 ${}^{a}$ ± 3.49	32.04 ${}^{a}$ ± 3.92
WtH (cm)	113.55 ${}^{a}$ ± 4.31	117.50 ${}^{b}$ ± 7.05	116.07 ${}^{b}$ ± 6.07	128.13 ${}^{c}$ ± 5.06	126.99 ${}^{c}$ ± 5.49	133.34 ${}^{d}$ ± 5.25	114.98 ${}^{b}$ ± 5.5	116.15 ${}^{b}$ ± 5.2
HtGC (cm)	155.15 ${}^{a}$ ± 9.59	156.94 ${}^{a}$ ± 11.49	144.88 ${}^{b}$ ± 10.46	158.67 ${}^{a}$ ± 9.52	170.39 ${}^{c}$ ± 9.87	172.92 ${}^{d}$ ± 8.69	147.98 ${}^{b}$ ± 8.36	146.63 ${}^{b}$ ± 9.15
BdL (cm)	111.32 ${}^{a}$ ± 6.72	110.46 ${}^{a}$ ± 8.04	129.33 ${}^{b}$ ± 7.34	136.42 ${}^{c}$ ± 7.58	129.37 ${}^{d}$ ± 8.39	130.52 ${}^{b}$ ± 12.87	106.86 ${}^{d}$ ± 9.41	114.37 ${}^{e}$ ± 8.38
HtG (cm)	61.81 ${}^{a}$ ± 2.58	59.54 ${}^{b}$ ± 3.54	56.05 ${}^{c}$ ± 4.18	60.78 ${}^{d}$ ± 3.4	67.98 ${}^{e}$ ± 4.71	64.59 ${}^{f}$ ± 4.71	55.34 ${}^{c}$ ± 4.11	58.05 ${}^{g}$ ± 3.28
HpH (cm)	114.11 ${}^{a}$ ± 4.63	121.18 ${}^{b}$ ± 6.11	118.44 ${}^{c}$ ± 5.31	134.62 ${}^{d}$ ± 5.34	134.00 ${}^{d}$ ± 5.53	139.43 ${}^{e}$ ± 4.90	119.34 ${}^{c}$ ± 5.56	118.47 ${}^{c}$ ± 4.58
RmL (cm)	36.98 ${}^{a}$ ± 2.29	32.88 ${}^{b}$ ± 4.50	34.85 ${}^{c}$ ± 2.98	45.66 ${}^{d}$ ± 2.73	34.32 ${}^{c}$ ± 3.45	32.48 ${}^{b}$ ± 3.40	28.66 ${}^{e}$ ± 7.08	36.04 ${}^{a}$ ± 6.76
HpW (cm)	34.70 ${}^{a}$ ± 2.16	38.65 ${}^{b}$ ± 2.79	25.34 ${}^{c}$ ± 2.84	42.10 ${}^{d}$ ± 3.68	46.84 ${}^{e}$ ± 3.94	44.34 ${}^{f}$ ± 3.67	36.78 ${}^{g}$ ± 2.85	36.88 ${}^{g}$ ± 2.88
RmW (cm)	11.57 ${}^{a}$ ± 1.40	11.64 ${}^{a}$ ± 1.59	11.76 ${}^{a}$ ± 1.32	13.58 ${}^{b}$ ± 1.61	13.86 ${}^{b}$ ± 2.00	13.89 ${}^{b}$ ± 1.32	11.84 ${}^{a}$ ± 1.37	10.99 ${}^{c}$ ± 1.04
TiL (cm)	68.82 ${}^{a}$ ± 4.82	82.79 ${}^{b}$ ± 7.32	81.00 ${}^{b}$ ± 5.85	99.04 ${}^{c}$ ± 6.92	90.98 ${}^{d}$ ± 9.10	99.94 ${}^{c}$ ± 7.18	79.19 ${}^{e}$ ± 7.94	83.70 ${}^{b}$ ± 6.20
TiLS (cm)	93.02 ${}^{a}$ ± 6.94	110.46 ${}^{b}$ ± 9.62	100.57 ${}^{c}$ ± 7.05	130.67 ${}^{d}$ ± 7.64	121.86 ${}^{e}$ ± 13.65	128.25 ${}^{f}$ ± 8.77	105.10 ${}^{g}$ ± 11.5	111.55 ${}^{b}$ ± 10.59
BW (kg)	253.16 ${}^{a}$ ± 41.75	309.04 ${}^{b}$ ± 61.53	211.86 ${}^{c}$ ± 66.42	333.70 ${}^{d}$ ± 51.98	412.26 ${}^{e}$ ± 76.64	412.76 ${}^{f}$ ± 70.06	261.94 ${}^{a}$ ± 36.36	259.18 ${}^{a}$ ± 39.11

Different superscript letters in the same row indicate a statistically significant difference between means ( P<0.05 ). n is the cow number, HdL is the head length, HdW is the head width, HrL is the horn length, HrC is the horn circumference, ErL is the ear length, ErW is the ear width, CsW is the chest width, WtH is the withers height, HtGC is the heart girth circumference, BdL is the body length, HtG is the heart girth, HpH is the hip height, RmL is the rump length, HpW is the hip width, RmW is the rump width, TiL is the tail length, TiLS is the tail length up to switch, and BW is the body weight.

**Table 3:** Physical characteristics of Indonesian cattle.

Breeds	FCo	DSz	HmSz	HrCo	HrOr	SCo	MSp	MCo	CCo	HoCo	HoSp	VCo
Bali	100 % brick red	100 % small	100 % absent	100 % black	Varied	100 % black	11.03 % moderately sharp, 88.97 % flat	100 % black	100 % brick red	100 % black	100 % sharp	100 % black
Rambon	Varied	100 % small	88 % absent, 12 % small	98 % black, 1 % gray, 1 % black–white	Varied	Varied	2 % moderately sharp, 98 % flat	100 % black	Varied	100 % black	100 % sharp	Varied
Madura	100 % red	100 % small	100 % small	100 % black	Varied	100 % black	94.70 % flat, 5.30 % sharp	100 % black	100 % red	100 % black	100 % sharp	100 % black
Ongole Grade	Varied	2.11 % large, 51.41 % medium, 46.48 % small	17.61 % medium, 82.39 % small	96.48 % black, 3.52 % brown	Varied	97.18 % black, 1.41 % brown, 0.70 % white–black, 0.70 % white	83.10 % flat, 16.90 % sharp	94.37 % black, 5.63 % pale	95.77 % white, 4.23 % beige	100 % black	100 % sharp	48.59 % black, 51.41 % pale
Sasra	83.93 % black, 1.79 % black–red, 14.29 % black–white	21.43 % medium, 78.57 % small	100 % absent	68.75 % absent, 31.25 % black	Varied	100 % black	100 % flat	100 % black	100 % black	100 % black	100 % sharp	100 % black
Kebumen Ongole Grade	100 % white	15.57 % large, 79.64 % medium, 4.79 % small	23.35 % large, 71.86 % medium, 4.79 % small	95.81 % black, 4.19 % black–white	Varied	89.22 % black, 10.78 % black–white	100 % flat	70.66 % black, 29.34 % pigmented	100 % white	100 % black	95.21 % sharp, 4.79 % moderately sharp	49.10 % black, 50.90 % pale
Jabres	Varied	30.94 % medium, 69.06 % small	8.63 % medium, 91.37 % small	97.12 % black, 1.44 % brown, 1.44 % white	Varied	Varied	99.28 % flat, 0.72 % moderately sharp	96.40 % black, 2.88 % pigmented, 0.72 % pale	Varied	97.12 % black, 1.44 % brown, 1.44 % white	100 % sharp	Varied
Pasundan	Varied	100 % small	100 % absent	99.02 % black, 0.98 % red	Varied	Varied	100 % flat	100 % black	Varied	100 % black	100 % sharp	Varied

FCo is the forehead coat color, DSz is the dewlap size, HmSz is the hump size, HrCo is the horn color, HrOr is the horn orientation, SCo is the switch color, MSp is the muzzle shape, MCo is the muzzle color, CCo is the coat color, HoCo is the hoof color, HoSp is the hoof shape, and VCo is the vulva color.

**Figure 3:**
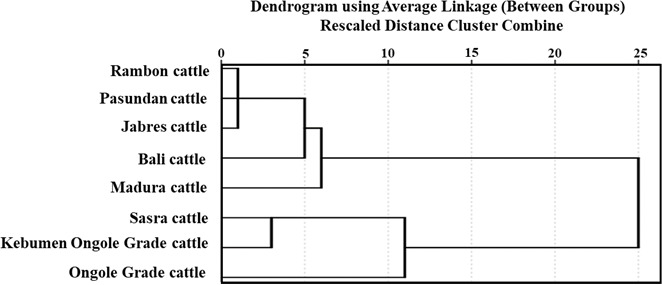
Dendrogram of the investigated Indonesian cattle.

**Figure 4:**
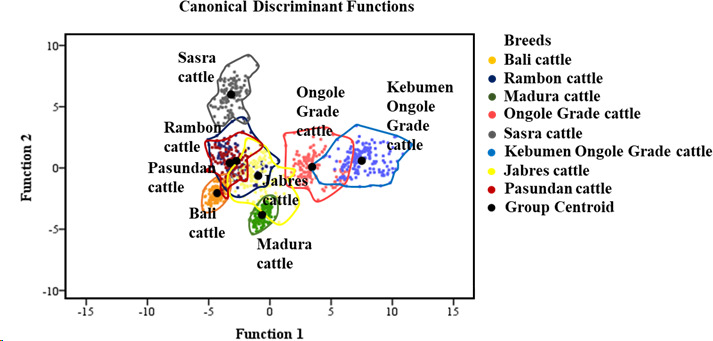
Classification of Indonesian cattle based on a canonical discriminant function.

Table 4 shows the genetic distances among breeds. The Rambon and Pasundan cattle breeds were in closest proximity (proximity value = 2.014), while the genetic distance was greatest between Bali and Kebumen Ongole Grade cattle (proximity value = 8.109). The above-described dendrogram depicting the two Indonesian cattle clusters is shown in Fig. 3. The first cluster included Jabres, Pasundan, Rambon, Madura, and Bali cattle, while the second included Ongole Grade, Kebumen Ongole Grade, and Sasra cattle. Figure 4 shows the results of a canonical discriminant analysis; most breeds formed distinct groups, except for the Rambon and Pasundan cattle, which overlapped. Table 5 provides the percentage values for the individual classifications, which were used to better understand the results of the discriminant analysis.

**Table 4:** The Euclidean distances between cattle populations.

Breeds	Bali	Rambon	Madura	Ongole	Sasra	Kebumen	Jabres	Pasundan
				Grade		Ongole Grade		
Bali	0.000							
Rambon	2.689	0.000						
Madura	3.506	3.471	0.000					
Ongole Grade	6.888	5.335	6.381	0.000				
Sasra	7.149	5.502	7.586	4.182	0.000			
Kebumen Ongole Grade	8.109	6.175	8.086	3.811	2.585	0.000		
Jabres	3.130	2.225	3.030	6.879	7.353	7.866	0.000	
Pasundan	3.006	2.014	2.516	5.716	6.799	7.405	2.137	0.000

**Table 5:** Percentage of individual classifications in the cattle breed populations (%).

Breeds	Bali	Rambon	Madura	Ongole	Sasra	Kebumen	Jabres	Pasundan
				Grade		Ongole Grade		
Bali	100.00	–	–	–	–	–	–	–
Rambon	5.00	57.00	–	–	2.00	–	12.00	24.00
Madura	–	–	100.00	–	–	–	–	–
Ongole Grade	–	–	–	99.30	–	0.70	–	–
Sasra	–	–	–	–	100.00	–	–	–
Kebumen Ongole Grade	–	–	–	1.20	–	98.80	–	–
Jabres	–	2.16	1.44	2.16	–	–	94.24	–
Pasundan	1.96	13.73	–	–	–	–	–	84.31

Table 6 shows the BW estimation models. The Bali cattle population had the highest $R^{2}$ (0.84) and lowest RMSE (15.98), while Kebumen Ongole Grade cattle had the lowest $R^{2}$ (0.37) and highest RMSE (54.87). The morphometric character HtGC was a component of all equations. Morphometric indices were also estimated and are summarized in Table 7. A negative HSI value indicates that the front of the body is lower than the back; the lowest HSI value was seen in Sasra cattle and the highest in Bali cattle. WSI represents the relative proportions of front width (positive value <1 ) and back width (positive value >1 ) values close to or equal to 1 for a given breed. Madura cattle had a higher proportion of positive front width values, while the other cattle types had a higher proportion of positive back width values. Overall, Jabres cattle had the best values. The LI values for Sasra, Ongole Grade, and Madura cattle were >1 (long type), while those for all other breeds were <1 (tall type). The DI values of Bali, Rambon, Sasra, and Pasundan cattle were >0.5 (short-legged type), while those for all other breeds were <0.5 (long-legged type). The FLI values indicated that Kebumen Ongole Grade had the longest forelegs and Bali cattle the shortest. Furthermore, Ongole Grade cattle had the highest B and CI values.

**Table 6:** Models for body weight estimation of Indonesian cattle.

Breeds	$R^{2}$	RMSE	Equations
Bali	0.84	15.98	Y=-527.644+3.350HtGC+0.915BdL+2.523RmW-1.899ErL+2.370HdL
			+0.447TiLS+1.018HpW
Rambon	0.44	44.98	Y=-410.917+1.984HtGC+5.08HtG+7.527ErW
Madura	0.38	51.60	Y=-602.977+2.093HtGC+2.633BdL+3.055HtG
Ongole Grade	0.69	28.47	Y=-557.232+3.220HtGC+6.218RmW+2.487CsW+0.928TiL+0.861BdL
Sasra	0.41	57.72	Y=-562.017+2.721HtGC+15.492RmW+5.085RmL
Kebumen Ongole Grade	0.37	54.87	Y=-866.848+3.271HtGC+3.057HpH+8.642RmW+2.798CsW
Jabres	0.71	19.31	Y=-260.312+3.504HtGC+5.614ErW-1.709HdL
Pasundan	0.30	32.28	Y=-178.419+1.702HtGC+3.238HtG

$R^{2}$ is the coefficient of determination, RMSE is the root mean squared error, HtGC is the heart girth circumference, HtG is the heart girth, BdL is the body length, RmW is the rump width, RmL is the rump length, ErW is the ear width, ErL is the ear length, HdL is the head length, HpW is the hip width, HpH is the hip height, CsW is the chest width, TiL is the tail length, and TiLS is the tail length up to switch.

**Table 7:** Morphological indices of Indonesian cattle.

Breeds	HSI	WSI	LI	DI	FLI	B	CI
Bali	- 0.56 ± 2.84	1.11 ± 0.10	0.98 ± 0.04	0.55 ± 0.03	51.73 ± 4.10	0.66 ± 0.08	2.46 ± 0.16
Rambon	- 3.68 ± 6.64	1.18 ± 0.14	0.94 ± 0.08	0.51 ± 0.04	57.96 ± 6.97	0.65 ± 0.12	2.59 ± 0.21
Madura	- 2.37 ± 4.87	0.83 ± 0.11	1.12 ± 0.07	0.48 ± 0.04	60.02 ± 6.00	0.52 ± 0.08	2.32 ± 0.25
Ongole Grade	- 6.49 ± 3.83	1.22 ± 0.12	1.07 ± 0.05	0.48 ± 0.03	67.35 ± 4.85	0.92 ± 0.11	3.06 ± 0.22
Sasra	- 7.01 ± 6.11	1.15 ± 0.14	1.02 ± 0.06	0.54 ± 0.04	59.01 ± 6.39	0.58 ± 0.10	2.93 ± 0.28
Kebumen Ongole Grade	- 6.08 ± 5.58	1.20 ± 0.15	0.98 ± 0.10	0.49 ± 0.04	68.76 ± 6.74	0.60 ± 0.10	2.92 ± 0.27
Jabres	- 4.36 ± 4.38	1.02 ± 0.12	0.93 ± 0.07	0.48 ± 0.03	59.64 ± 4.53	0.62 ± 0.13	2.39 ± 0.22
Pasundan	- 2.32 ± 5.15	1.17 ± 0.16	0.99 ± 0.07	0.50 ± 0.03	58.10 ± 5.03	0.73 ± 0.17	2.55 ± 0.23

HSI is the height slope index, WSI is the width slope index, LI is the length index, DI is the depth index, FLI is the foreleg length index, B is the balance, and CI is the cumulative index.

## 4 Discussion

The morphometric differences among cattle breeds observed in our study (Table 2) might be attributable to environmental and breed factors. Previously, a strong relationship between morphometrics and production potential was reported (Goitom et al., 2019). The values of HdL, HdW, ErL, ErW, HrL, and HrC reflect the appearance of the face and head of an animal. These values are very important for distinguishing among breeds; in fact, they are the basis on which different livestock breeds are generated. Furthermore, meat-related morphometric measures such as RmL, CsW, HtGC, and HpW can be used as indices of production performance and to characterize livestock populations. These indices allow the assessment, management, and conservation of livestock based on morphological relationships (Bousbia et al., 2021). A larger body frame can support a larger body, where body size is associated with the structure of body components. BW is a proxy for body size (Babale et al., 2018).

Physical characteristics must be considered in livestock breeding programs. In our study, the orientation of the horns varied among the animals and had no bearing on breeding activities (Table 3). Physical characteristics are critical for cattle identification and morphometric measurements and serve as basic data for breeding programs (Bhinchhar et al., 2017). The unique characteristics of individual breeds can provide a foundation for breeding programs, although it is often difficult to distinguish cattle breeds based on physical characteristics because of the high within-breed variation (Moussa et al., 2017). Interestingly, in Sasra cattle, a local breed in Indonesia that has the potential to be maintained, the percentages of horned and hornless cattle were 31.25 % and 68.75 %, respectively. Sasra cattle with horns are thought to be the result of past random mating. These black cattle were developed after the introduction of artificial insemination technology (1976–1982) at the Lembang Artificial Insemination Center, using frozen semen from Aberdeen Angus cattle (Adinata et al., 2017; Subiharta et al., 2021). Aberdeen Angus cattle have black coats and are naturally polled (Morgan, 2021). The recipient cows were mostly Ongole Grade cattle. The horned trait in cattle appears in homozygous recessives, while the polled character is dominant (Gehrke et al., 2020).

The genetic closeness between Rambon and Pasundan seen in this study, and the wide gap between Bali and Kebumen Ongole Grade cattle, might be due to their morphological appearance (Dogan and Dogan, 2016). Figure 1 clearly shows that Rambon and Pasundan cattle appear similar but have distinct breeding ranges, while Bali and Kebumen Ongole Grade cattle differ in both appearance and distribution area. The morphometric distances (Table 4) and clustering (Fig. 3) results of this study are consistent with the results of phylogenetic analyses of various types of molecular data (Agung et al., 2019; Prihandini et al., 2020; Sudrajad et al., 2020). Phylogenetic diversity is the best objective criterion for conserving and protecting taxonomically different breeds through genetic resource conservation programs (Brooks et al., 2015).

The discriminant function analysis revealed overlap between the Rambon and Pasundan cattle populations (Fig. 4); Table 5 shows this in more detail. Based on cross-validation, only individuals from the Bali, Madura, and Sasra cattle breeds were grouped 100 % correctly; the average suitability value for the remaining breeds was 93.20 %. Even when there is some overlap between centroids, differentiation between breeds is possible (Navas et al., 2021).

Based on the equation shown in Table 6, the HtGC is an important quantitative measure for estimating animal BW. However, other variables not included in the equation must also be considered. The estimation of the relationship between $R^{2}$ and RMSE in this study was not very accurate; the trend of increasing $R^{2}$ values was not mirrored by the trend of decreasing RMSE values. Higher positive B index values indicates higher meat production potential; in this study, Ongole Grade and Madura cattle had the highest and lowest values, respectively (Table 7). Cattle with higher CI values are considered superior in terms of growth rate and quality; Ongole Grade cattle had the highest CI values in this study. Because the CI value is related to age, it can be used to predict growth rates. Ongole Grade cattle with CI values >3 can be used as a basis for selection programs; this breed was developed as a beef producer and draft animal (Sutarno and Setyawan, 2016; Sudrajad et al., 2020).

## 5 Conclusion

The Indonesian cattle in this study formed two distinct clusters. The first cluster consisted of Rambon, Jabres, and Pasundan cattle and had an average suitability value of 93.20 %; the second cluster consisted of Bali, Madura, and Sasra (average suitability value = 100 %). These classification results will help distinguish cattle breeds. The most important morphometric trait for estimating BW is the HtGC. A cumulative index >3 can be used as a threshold for determining the type and function of beef cattle. Ongole Grade cattle were estimated to have better growth rates and could be used as potential beef cattle in Indonesia.

## Data Availability

The datasets analyzed during the current study are available from the corresponding author on reasonable request.
